# Mechanistic Resolution Required to Mediate Operant Learned Behaviors: Insights from Neuronal Ensemble-Specific Inactivation

**DOI:** 10.3389/fncir.2017.00028

**Published:** 2017-04-24

**Authors:** Brandon L. Warren, Nobuyoshi Suto, Bruce T. Hope

**Affiliations:** ^1^Behavioral Neuroscience Branch, Intramural Research Program (IRP), National Institute on Drug Abuse (NIDA), National Institutes of Health (NIH), Department of Health and Human Services (DHHS)Baltimore, MD, USA; ^2^Department of Molecular and Cellular Neuroscience, The Scripps Research InstituteLa Jolla, CA, USA

**Keywords:** learned associations, neuronal ensembles, mechanistic resolution, operant learning

## Abstract

Many learned behaviors are directed by complex sets of highly specific stimuli or cues. The neural mechanisms mediating learned associations in these behaviors must be capable of storing complex cue information and distinguishing among different learned associations—we call this general concept “mechanistic resolution”. For many years, our understanding of the circuitry of these learned behaviors has been based primarily on inactivation of specific cell types or whole brain areas regardless of which neurons were activated during the cue-specific behaviors. However, activation of all cells or specific cell types in a brain area do not have enough mechanistic resolution to encode or distinguish high-resolution learned associations in these behaviors. Instead, these learned associations are likely encoded within specific patterns of sparsely distributed neurons called neuronal ensembles that are selectively activated by the cues. This review article focuses on studies of neuronal ensembles in operant learned responding to obtain food or drug rewards. These studies suggest that the circuitry of operant learned behaviors may need to be re-examined using ensemble-specific manipulations that have the requisite level of mechanistic resolution.

## Introduction

Learned associations between reinforcers and predictive stimuli are critical for survival. Unfortunately, these learned associations are also implicated in addictive behaviors. Indeed, part of what makes addictive behaviors so intractable is that craving and relapse are often triggered by exposure to closely associated stimuli (Wikler, 1973; Goldberg, 1975; O’Brien et al., 1984; Stewart et al., 1984; Siegel, 1999). Likewise, exposure to predictive stimuli causes reinstatement of both drug and natural reward seeking in animal models (Davis and Smith, 1976; de Wit and Stewart, 1981; Weingarten, 1983; Petrovich et al., 2007; Yager and Robinson, 2010). Furthermore, the predictive stimuli that trigger relapse can be very specific (Powell et al., 1990). Thus, memories underlying this type of high-resolution behavior must be represented within the brain with equally high-fidelity mechanistic resolution. Mechanistic resolution describes the physical properties of the brain that underlie its resolving power. The brain’s ability to encode and distinguish among different sets of highly specific stimuli necessitates high mechanistic resolution. Because the brain can convert compound cues and contexts into highly specific memories, this conversion process must be able to translate highly specific information into physiological changes within the brain.

Currently, the majority of studies attempting to investigate the neurobiology of memory assess “global” alterations and their functions within brain regions, neurons of a specific cell-type, or afferent/efferent connections between two brain areas, regardless of the specific neural activity patterns during behavior (Nestler et al., 1993; Kalivas et al., 2005; Hyman et al., 2006; Koob, 2006; Bowers et al., 2010; Jennings et al., 2013; Otis et al., 2017). These experiments have traditionally relied upon pharmacological inactivation of discrete regions or receptor subtypes. More recently, optogenetic and chemogenetic approaches have allowed for more precise targeting of brain regions, circuits, or cell types and have given us greater insight into their role in memory than ever before. However, the view that global alterations within a given brain area can represent each unique learned association between a distinct environmental cue and a specific behavioral response is incompatible with the characteristics of mechanistic resolution. An alteration that effects all cells of a given type or within a given brain area or circuit can be thought of as a binary change. In this case, each cell would be affected similarly, which means there are only two possible states (on or off). Thus, the mechanistic resolution of a global alteration is limited to two behavioral states, making it an unlikely mechanism for storing and distinguishing among specific memories. Instead, high-resolution information is thought to be encoded within specific patterns of neurons, called neuronal ensembles, that are selectively activated in response to specific cues and reinforcers during behavior.

## Fos-Expressing Neuronal Ensembles are Capable of High Mechanistic Resolution

**Figure 1:**
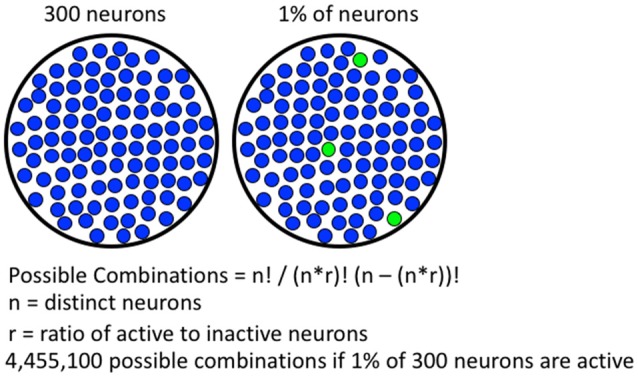
**Formula for calculating number of possible combinations of activated neurons.** A hypothetical brain region containing 300 neurons is shown on the left, if 1% of the neurons are active, 4,455,100 possible combinations are possible. With more neurons available in real brain areas, the number of possible combinations is much greater.

Immediate early gene (IEG) expression is an indirect marker used to assess neuronal activity (Cruz et al., 2015). The most commonly used IEG, Fos, can be detected within 10–30 min of strong neuronal activity. The Fos promoter is activated when strong and persistent calcium influx coincides with high levels of excitatory input, leading to expression of Fos mRNA and protein (Morgan and Curran, 1991; Deisseroth et al., 2003; Cohen and Greenberg, 2008; Brami-Cherrier et al., 2009; Cahill et al., 2014; Kawashima et al., 2014); the extensive literature is described in more detail in Cruz et al. (2015). It is thought that the neurons receiving highest glutamatergic input are selected by the cues and contexts present during self-administration (Cruz et al., 2015). In aggregate, these activated neurons make up the group of neurons that form a neuronal ensemble. Fos-expressing neuronal ensembles meet the requirements for mechanistic resolution. Specifically, Fos-expressing neuronal ensembles are capable of representing highly specific associations based on patterns of activation and connectivity. Studies indicate about 5% or less of neurons in a histochemical section are Fos-expressing following most operant learned behaviors (Bossert et al., 2011; Fanous et al., 2013; Warren et al., 2016; Caprioli et al., 2017). These percentages of Fos-expressing neurons, quantified in two-dimensional histochemical slices, translate to less than 1% of neurons in three-dimensional brain volumes. Since less than 1% of neurons in a brain volume are activated enough to express Fos, there is an immense number of possible patterns for Fos-expressing neuronal ensembles (see Figure 1). Furthermore, each learned association is thought to be represented by a specific pattern of Fos-expressing neurons in each brain region. The immense number of possible configurations (or neuronal ensembles) allows for many distinct associations to be made and stored within a single brain region, which provides the high degree of mechanistic resolution required to encode learned associations among complex sets of high resolution cues, contexts and rewards in operant learned behaviors.

Work using single cell electrophysiology appears to support this Fos-expressing neuronal ensemble hypothesis. Within the nucleus accumbens, single unit recordings during operant responding have demonstrated that different rewards cause firing in different neurons (Carelli and Deadwyler, 1994; Chang et al., 1998; Carelli, 2002a; Carelli and Wondolowski, 2003; Deadwyler et al., 2004; Opris et al., 2009; Cameron and Carelli, 2012). Neurons activated by one reinforcer (cocaine) are often not activated by a second reinforcer (food; Carelli and Ijames, 2001; Carelli, 2002b). This suggests that the two learned associations activate largely different populations of neurons, likely different neuronal ensembles. However, evidence that distinct Fos-expressing neuronal ensembles co-mingle within the same brain area and play causal roles in distinct learned associations has been lacking.

## Fos-Expressing Neuronal Ensembles Mediate Operant Learned Behaviors

**Figure 2:**
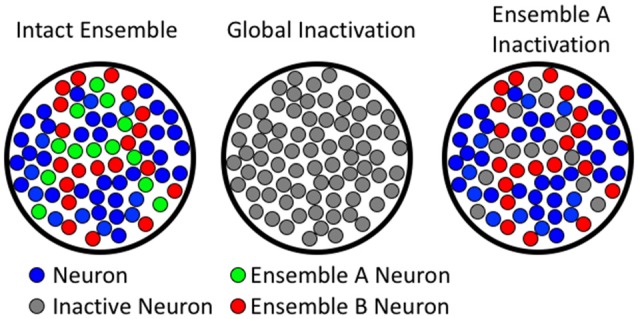
**Comparing global to ensemble-specific inactivation.** The left column shows hypothetical neuronal ensembles. The middle column illustrates that global inactivation inactivates all neurons (both ensemble A and B). The right column shows ensemble-specific inactivation of ensemble A, leaving ensemble B unaffected.

Current methods for demonstrating whether a brain region or circuit is necessary for behavior rely on technologies that inactivate either a whole brain region, or specific phenotypes of neurons (e.g., glutamatergic vs. GABAergic projecting neurons), without accounting for differences in neuronal activity. While these technologies have provided a strong framework for our understanding of the neuroanatomy of behavior, they are not congruent with the processing, storage, or discrimination of high-resolution information in learned behaviors (see Figure 2). Several technologies have recently emerged that enable neuronal ensembles to be labeled and manipulated *in vivo*; these technologies have previously been described in Mayford et al. (1996); Cruz et al. (2013); Kawashima et al. (2013, 2014); Ramirez et al. (2013); Liu et al. (2014); and Sørensen et al. (2016). This review, however, focuses on the biological question of how Fos-expressing neuronal ensembles can mediate operant learned responding for food and drug rewards with high mechanistic resolution. We provide evidence that two of these ensembles can intermingle in the same brain area to encode separate learned associations that drive behavior in opposing directions. We also describe how low resolution global manipulations of neural activity (excitation or inhibition) cannot distinguish these ensembles, and even mask the multiple roles of a brain area in the behavior of interest.

## Inactivating Fos-Expressing Ensembles using the Daun02 Chemogenetic Procedure

The earliest technology used to demonstrate a causal role for behaviorally activated Fos-expressing neuronal ensembles in a learned behavior was the Daun02 inactivation procedure using Fos-LacZ transgenic rats (Bossert et al., 2011; Cruz et al., 2013, 2014, 2015; Koya et al., 2016). Indeed, the Daun02 inactivation procedure has been used in the vast majority of studies that examined Fos-expressing ensembles in operant learned behaviors. In Fos-lacZ rats, activation of the Fos promoter leads to expression of the LacZ coding sequence, resulting in translation of Fos as well as the enzyme β-galactosidase (βgal) only in strongly activated neurons. Following βgal induction, the inactive prodrug Daun02 is infused into the brain region of interest. Daun02 is catalyzed by βgal into daunorubicin, a toxic compound that leads to early inactivation of calcium-dependent action potentials (Engeln et al., 2016), followed by apoptosis and cell death only in the strongly activated βgal-expressing neurons (Pfarr et al., 2015). After a couple of days, the behavioral effects of the targeted neural inactivation by Daun02 can be assessed under a drug free condition (Bossert et al., 2011; Fanous et al., 2012; Koya et al., 2016) to demonstrate causal roles for neuronal ensembles within multiple nodes of the corticostriatal circuit, including prefrontal cortex, striatum and amygdala (Bossert et al., 2011; Cruz et al., 2013, 2014, 2015; Pfarr et al., 2015; de Guglielmo et al., 2016; Funk et al., 2016; Koya et al., 2016; Suto et al., 2016; Caprioli et al., 2017).

In 2009, our group published a series of experiments designed to assess a role for neuronal ensembles in the environmental control of sensitized locomotor responding for cocaine (Koya et al., 2009). We administered cocaine to rats in a specific environmental context in daily sessions for 7 days. In a subsequent test, rats are given a threshold dose of cocaine and placed in either a novel context, or into the context that was previously paired with cocaine. Only rats placed in the context previously paired with cocaine showed hyperlocomotion (Koya et al., 2009). This difference represents a learned association between the effects of cocaine and the environmental context. Rats that were re-exposed to the cocaine-paired context also have increased Fos-expression within the nucleus accumbens. We then used the Daun02 inactivation procedure to test whether these Fos-expressing neuronal ensembles played a causal role in maintaining the learned association between cocaine’s effects and the context. Inactivation of neuronal ensembles associated with the cocaine administration context significantly blunted sensitized cocaine-induced locomotion. This finding suggested that Fos-expressing neuronal ensembles do play a causal role in cocaine sensitization, despite representing less than 1% of neurons. Unlike ensemble-specific lesions of behaviorally activated Fos-expressing neurons, lesions of the nucleus accumbens brain region paradoxically enhanced locomotor sensitization for both nicotine and cocaine (Kelsey and Willmore, 2006).

Subsequent studies also indicate very different effects on operant learned behavior when using global inactivation methods vs. ensemble-specific inactivation methods. Bossert et al. (2011) demonstrated that Fos-expressing neuronal ensembles mediate context-induced reinstatement of heroin seeking. Rats were first trained to lever press for heroin infusions in one context (context A), then the behavior was extinguished in a second context (context B). Under normal conditions, re-exposure to context A induces reinstatement of heroin seeking. The authors show that re-exposure to context A induced robust Fos-expression within the vmPFC. Daun02 inactivation of neurons associated with exposure to the heroin context significantly reduced context-induced reinstatement of heroin seeking (Bossert et al., 2011). This strongly suggests that neuronal ensembles within the vmPFC mediate learned associations about the heroin-context. An ancillary finding was that a separate group of rats, exposed to the extinction context prior to Daun02 administration had somewhat higher responding in a subsequent test of extinction recall. Although this increase was not significant (likely due to limited numbers of rats in each group), it suggested that the infralimbic cortex might also be involved in extinction. This is particularly interesting because global inactivation of the infralimbic cortex paradoxically increased cocaine seeking behavior in extinguished rats (Peters et al., 2008), suggesting that this brain area is involved in extinction (although others have found contradictory findings in cue-induced reinstatement of heroin seeking (Rogers et al., 2008), and methamphetamine seeking (Rocha and Kalivas, 2010)). Nevertheless, these results provided the first hint that two distinct memories (self-administration and extinction) could be represented separately in the vmPFC.

To more directly test this hypothesis, we used self-administration of palatable food pellets to show that self-administration and extinction memories are represented separately within the vmPFC (Warren et al., 2016). We first trained rats to self-administer palatable food pellets in daily sessions. Once reliable responding was achieved, we exposed rats to three different extinction conditions (0, 2, or 7 daily extinction sessions). Fos expression in the vmPFC was highest following 2 days of extinction, suggesting a period of enhanced neuronal ensemble formation, likely corresponding to formation of the extinction memory. We hypothesized that rats undergoing 0 days of extinction would retain the intact self-administration memory, while rats undergoing 2 days of extinction would have formed an extinction memory in addition to the self-administration memory. Global inhibition of the vmPFC did not influence responding for palatable food pellets in either group. This contrasts with earlier work that used global inactivation of vmPFC to inhibit context-induced reinstatement for food-seeking and induce reinstatement of food-seeking in the extinction context (Eddy et al., 2016). Despite the finding that global inactivation of the vmPFC had no effect on behavior, we found that selective inactivation of neuronal ensembles associated with self-administration impaired self-administration recall. Conversely, inactivation of neuronal ensembles associated with extinction impaired extinction recall. This suggests that both self-administration and extinction ensembles intermingle within the vmPFC. Perhaps more astonishing was the finding that region-wide inactivation did not influence behavior, while ensemble-specific inactivation with the Daun02 procedure did. This disparity underscores a fundamental flaw present in global inactivation studies. In this experiment, region-wide inactivation simply did not have the technical resolution required to disentangle these competing ensembles. This may also be one reason that earlier experiments using region-wide manipulations did not see differences in food-seeking. The key is that the difference is in *which* cells are inactivated, rather than *how many* or *what kind* of cells are inactivated.

The distinction between *which* cells and *how many* cells influence behavior was recently tested using a new line of transgenic rats (Pfarr et al., 2015). The pCAG-LacZ line of transgenic rats express βgal ubiquitously under the control of the pCAG promoter. Thus, by comparing results of Daun02 inactivation in Fos-LacZ (activity dependent inactivation) and pCAG-LacZ (non-selective inactivation) transgenic rats it is possible to directly compare the effect of region-wide inactivation with selective activity-dependent inactivation. The first study using this methodology exposed a key difference between non-selective and selective activity-dependent inactivation of the vmPFC in cue-induced reinstatement of alcohol seeking (Pfarr et al., 2015). Inactivation of neuronal ensembles associated with alcohol-seeking using the Daun02 procedure in Fos-LacZ rats resulted in a profound increase in cue-induced alcohol seeking. These results appear to demonstrate that neuronal ensembles associated with alcohol cues inhibit alcohol seeking. While this study seems to contradict findings from our lab, it is likely that several methodological and pharmacological differences might explain this discrepancy. While resolving these issues is likely outside the scope of this review, there is one clear take-away that unites these studies. Importantly, the authors found that inactivation of the entire vmPFC in pCAG-LacZ rats had no effect on cue-induced alcohol seeking. Thus, activity-dependent inactivation of a small number of neurons had a significant effect on behavior, while global inactivation had no effect.

Perhaps the most compelling evidence for the importance of selectively inactivating *which* rather than *how many* or *what kind* of cells comes from another study (Suto et al., 2016). In this study, the rats were first operant conditioned to self-administer a sweet solution (containing both glucose and saccharine). Lever insertion signaled onset of each self-administration session and each lever press led to cue-light illumination. The rats were then trained to recognize two distinct auditory cues (white noise and beeping tone) as the discriminative stimulus predictive of reward availability (S+) or reward omission (S−). Subsequent tests revealed that S+ and S−, respectively, potentiated and suppressed basal reward seeking induced by lever and cue-light.

Daun02 was then used to disrupt infralimbic cortical neurons selectively reactive to S+ or S−. Following recovery, rats were tested again for the bidirectional modulation of reward seeking by S+ and S−. Inactivation of S+ associated neurons blocked the promotion of reward seeking by S+, but spared the suppression by S−. In contrast, Daun02 lesion of S− associated neurons blocked the suppression of reward seeking by S− but spared the promotion by S+ (Suto et al., 2016). These findings provide the causal evidence for the concurrent existence of two distinct ensembles, each mediating opposing environmental actions on appetitive behavior, in the same vmPFC brain area. Like other studies reviewed here, these findings raise a caution to the use of non-selective techniques that manipulate neural activity irrespective of intrinsic neural activity for determining the brain-behavioral functions (Suto et al., 2016). Overall, these Daun02 inactivation studies indicate different effects on learned behaviors when using a high-resolution ensemble-specific manipulation as compared with low-resolution manipulations of whole brain areas or cell types.

## Inactivating Fos-Expressing Ensembles using Fos-tTA Transgenic Mice

As mentioned before, several other technologies have been developed to manipulate behaviorally activated neuronal ensembles in learned behaviors. These techniques have been used primarily to examine Pavlovian conditioned behaviors such as cue and/or context-induced fear conditioning (Mayford et al., 1996; Cruz et al., 2013; Kawashima et al., 2013, 2014; Ramirez et al., 2013; Liu et al., 2014; Sørensen et al., 2016). One of the most powerful approaches for identifying and manipulating these neuronal ensembles utilizes transgenic Fos-tTA (tetracycline(tet)-off transcriptional activator) mice from the Mayford lab (Reijmers et al., 2007; Wiltgen et al., 2007), and has been used to assess Fos-expressing neuronal ensembles in a number of learned behaviors, particularly fear conditioning (Reijmers et al., 2007; Garner et al., 2012; Liu et al., 2012, 2014; Ramirez et al., 2013; Redondo et al., 2014; Tonegawa et al., 2015; Yokose et al., 2017). Fos-tTA mice express tTA protein under the control of the Fos promoter so that only activated neurons express tTA protein. A second transgene with a tetracycline-responsive element promoter (TRE) allows tTA to drive expression of the second transgene only in activated neurons. The second transgene can be a fluorescent marker to identify activated neurons, a light-sensitive rhodopsin in optogenetic studies, or a DREADD receptor for chemogenetic studies. Importantly, the expression of the second transgene can be repressed by introducing doxycycline to the mouse’s diet, since doxycycline binds and represses tTA. Therefore, expression of the second transgene can be limited to a particular time window by maintaining doxycycline in the mouse’s diet, but removing doxycycline from the mouse’s diet prior to neuronal activation.

Fos-tTA mice have also been used to demonstrate that one can manipulate different Fos-expressing ensembles in the same brain area and induce opposing effects on behavior (Redondo et al., 2014). Different intermingling neuronal ensembles in the basolateral amygdala were activated during aversive or rewarding experience in conditioned place avoidance and preference assays. In these experiments, optogenetic activation of basolateral amygdala ensembles encoding the aversive or rewarding experience could be linked to previously neutral contextual stimuli stored in the hippocampus to induce conditioned place avoidance or preference, respectively. Separate intermingling neuronal ensembles in the basolateral amygdala have also been shown to encode both aversive and rewarding information and drive opposing effects on behavior (Kim et al., 2016). Overall, in animals that encode both aversive and rewarding unconditioned stimuli or learned associations in the same basolateral amygdala brain area, global inactivation (or activation) of both ensembles would have unpredictable net effects when these ensembles control the same behavior.

## Virus-Based Methods for Manipulating Neuronal Ensembles *In Vivo*

Viral based methods for manipulating neurons based on activity level represent another promising approach for identifying and manipulating neuronal ensembles. Several approaches have been proposed, although most have been limited to labeling activated neurons (Kawashima et al., 2014). Several of these methods now offer the ability to manipulate ensembles (Gore et al., 2015; Obenhaus et al., 2016; Sørensen et al., 2016). In particular, Gore et al. (2015) used a novel lentiviral system that employed a Fos promoter to drive expression of channelrhodopsin in Fos-expressing ensembles within the same basolateral amygdala brain area that encoded either aversive or rewarding unconditioned stimuli. Photoactivation of these two ensembles in the presence of previously neutral stimuli induced learned associations that induced opposing effects on behavior in response to the same cue, depending on which basolateral amygdala ensemble was activated during learning. Again, in animals that encode both aversive and rewarding unconditioned stimuli or learned associations in the same basolateral amygdala brain area, global inactivation (or activation) of both ensembles would have unpredictable net effects when these ensembles control the same behavior.

## Future Perspectives

Taken together, these findings raise questions about the utility of studies involving global manipulations—that alter (excite or inhibit) the activity of specific brain areas or circuits regardless of intrinsic neural activity—in directly mediating high-resolution operant learned behaviors. As shown, the low resolution of global manipulations can obscure a brain region’s role in behavior. Since environmental information and memory storage in the brain are more likely mediated by high-resolution mechanisms such as neuronal ensembles rather than by global alterations in whole brain areas, the ensemble-specific manipulations are much more congruent with how the brain processes, stores and distinguishes among high-resolution information in learned behaviors. Indeed, data is emerging that indicate selective molecular and cellular alterations in Fos-expressing neuronal ensembles in a variety of systems and behaviors. Pavlovian conditioned behaviors also appear to share a requirement for high mechanistic resolution mediated by Fos-expressing neuronal ensembles. As the technical resolution of our manipulations continues to advance, it is likely that we will gain an even more clear perspective on the role of neuronal ensembles in a variety of behaviors. In this way, technical resolution can more closely match the mechanistic resolution of the behavioral process being studied.

## Author Contributions

This is a mini review article. All authors researched and wrote the manuscript.

## Conflict of Interest Statement

The authors declare that the research was conducted in the absence of any commercial or financial relationships that could be construed as a potential conflict of interest.
